# Diagnostic value of PNI and MLR in cirrhotic patients with negative HBV DNA and positive HBV RNA:A nomogram--based evaluation model

**DOI:** 10.3389/fmed.2026.1919910

**Published:** 2026-10-19

**Authors:** Minying Li, Yan Fang, Yuhong Han, Tuantuan Li, Yan Liu, Lichang Chen

**Affiliations:** Department of Clinical Laboratory, the Second People's Hospital of Fuyang City, Fu Yang, China

**Keywords:** HBV DNA, HBV RNA, liver cirrhosis, MLR, PNI

## Abstract

**Introduction:**

To investigate the diagnostic significance of the Prognostic Nutritional Index (PNI) and the Monocyte-to-Lymphocyte Ratio (MLR) for the development of cirrhosis in patients with HBV DNA-negative, HBV RNA-positive hepatitis B, and to evaluate their clinical application value.

**Methods:**

A total of 87 patients with HBV DNA-negative, HBV RNA-positive hepatitis B who developed cirrhosis and were admitted to the patients from the Second People's Hospital of Fuyang City between March 2024 and March 2025 were enrolled as the observation group. Additionally, 96 patients with HBV DNA-negative and HBV RNA-positive hepatitis B during the same period were selected as the control group. Liver function, AFP levels, and five-part differential blood counts were measured in both groups. The PNI and MLR were calculated and compared between the two groups. ROC curves were plotted to analyze the sensitivity and specificity of PNI and MLR in diagnosing cirrhosis in patients with HBV DNA-negative, HBV RNA-positive hepatitis B, and their correlations were assessed. Furthermore, a nomogram model was established to evaluate the predictive ability of these two indicators for concomitant cirrhosis in this patient population, and the model was validated using a diagnostic calibration curve.

**Results:**

In No statistically significant differences were observed between the observation and control groups in terms of age, sex, ALT, ALP, or AFP levels (*P* > 0.05). Compared with the control group, patients in the observation group showed significantly lower levels of inflammatory markers (white blood cell, lymphocyte, and monocyte counts), nutritional markers (TP, ALB, PA), and PNI, while levels of AST, GGT and MLR were significantly higher (*P* < 0.05). A significant negative correlation was observed between PNI and MLR levels in the observation group (*r* = −0.484, *P* < 0.001). ROC curve analysis revealed that the areas under the curve of PNI and MLR for diagnosing cirrhosis in patients with HBV DNA-negative, HBV RNA-positive hepatitis B were 0.821 and 0.743, with sensitivities of 0.958 and 0.854, respectively (*P* < 0.001). The additional analyses convincingly demonstrate that PNI and MLR offer incremental clinical value beyond the widely used FIB-4 and APRI scores. Logistic regression analysis indicated that PNI overexpression was a protective factor for cirrhosis in this patient population. The calibration curve demonstrated good agreement between predicted and observed outcomes, and decision curve analysis suggested a positive net benefit for the clinical application of this model.

**Conclusion:**

The Prognostic Nutritional Index and the monocyte-to-lymphocyte ratio can serve as sensitive and effective biomarkers for the diagnosis of cirrhosis in patients with HBV DNA-negative, HBV RNA-positive hepatitis B. The established nomogram model provides a reference for the clinical diagnosis of cirrhosis in this patient population.

## 1 Introduction

Cirrhosis represents the terminal phase of chronic liver disease, pathologically defined by progressive diffuse hepatic fibrosis and the formation of regenerative nodules (pseudolobules), which disrupt the normal liver architecture (1). Clinically, it is characterized by progressive hepatic decompensation and clinically significant portal hypertension. Major life-threatening complications include rupture of gastroesophageal varices and overt hepatic encephalopathy (2). Both hepatitis B virus (HBV) DNA and RNA are employed as biomarkers to reflect the replicative activity of HBV. However, HBV RNA has been reported to effectively complement the assessment of viral replication status in patients who present with undetectable serum HBV DNA levels (3). A serological profile characterized by undetectable HBV DNA but detectable HBV RNA in patients with chronic hepatitis B or cirrhosis implies the persistence of active viral transcription and replication, albeit without demonstrable infectivity currently. Importantly, this status confers a sustained predisposition to virologic reactivation, underscoring the need for continued surveillance and cautious consideration of treatment withdrawal.

The Prognostic Nutritional Index (PNI) is a readily available and objective composite indicator calculated from serum albumin concentration and total lymphocyte count. Initially proposed as a prognostic factor for perioperative risk assessment, it has since been recognized as a reliable surrogate for the systemic nutritional and immune status of patients (4). More recently, the application of PNI has extended beyond its original scope, emerging as a composite clinical metric for the concurrent evaluation of nutritional status and long-term outcomes. Accumulating evidence has validated PNI as an independent prognostic factor in diverse pathological contexts, encompassing autoimmune diseases, cardiac and renal disorders, type 2 diabetes, and gastrointestinal tumors, underscoring its broad utility as a pan-disease prognostic tool (5–8). The monocyte-to-lymphocyte ratio (MLR), calculated as the quotient of monocyte count divided by lymphocyte count, has been widely adopted for assessing disease activity and predicting prognosis across various clinical conditions, owing to its straightforward calculation and low cost. As a composite indicator encompassing both innate and adaptive immunity, MLR reflects the systemic immuno-inflammatory status of patients (9). Both HBV DNA and RNA are recognized as critical diagnostic biomarkers in the management of chronic hepatitis B. They not only facilitate the accurate determination of infection phase but also provide objective measures for monitoring treatment efficacy and guiding therapeutic decisions (10). However, relatively few studies have investigated the diagnostic indicators for the development of cirrhosis in chronic hepatitis B patients with an undetectable HBV DNA but detectable HBV RNA profile. To address this knowledge gap, the present study was designed to investigate the utility of PNI and MLR as non-invasive biomarkers for the associate of cirrhosis in CHB patients exhibiting the HBV DNA-Negative/RNA-Positive serological profile. Additionally, we aimed to characterize the systemic immuno-inflammatory milieu in cirrhotic patients with ongoing viral transcription, as reflected by detectable HBV RNA despite DNA suppression. The findings of this study are expected to contribute to the early identification of at-risk patients and to inform evidence-based strategies for timely therapeutic intervention.

## 2 Materials and methods

### 2.1 Baseline characteristics

A total of 183 patients with chronic hepatitis B who were negative for HBV DNA but positive for HBV RNA were enrolled from the Second People's Hospital of Fuyang City between March 2024 and March 2025. Among them, 87 patients with cirrhosis were assigned to the observation group (49 males and 38 females, mean age 54.83 ± 9.57 years), and 96 patients without cirrhosis admitted during the same period were assigned to the control group. All patients received antiviral therapy, and the laboratory data were collected after treatment.

Inclusion criteria: (1) patients meeting the diagnostic criteria for chronic hepatitis B and cirrhosis as per established guidelines (11); (2) complete clinical data.

Exclusion criteria: (1) co-infection with other hepatitis viruses (hepatitis A, C, D, or E); (2) severe primary diseases involving the cardiovascular, cerebrovascular, renal, or hematopoietic systems; (3) concomitant malignancy; (4) incomplete clinical data; (5) coexisting autoimmune hepatitis, alcoholic liver disease, or other hepatic disorders.

Regarding the diagnosis of cirrhosis (Reference Standard):

In this study, the diagnosis of cirrhosis was established based on a combination of liver stiffness measurement (LSM) using transient elastography (Fibro Scan) and imaging findings, supplemented by clinical criteria when histological data were unavailable.

Specifically, cirrhosis was defined as LSM ≥ 12.0 kPa, in conjunction with ultrasonographic features suggestive of cirrhosis (e.g., nodular liver surface, blunt liver edge, and splenomegaly), and/or the presence of clinical signs of portal hypertension (e.g., thrombocytopenia [platelet count < 100 × 10⁹/L], or imaging-confirmed varices).

Liver biopsy was not used as the primary reference standard in this study because it was not routinely performed for all patients due to its invasive nature. However, in cases where biopsy was available, histological results were prioritized and showed good concordance with the non-invasive diagnostic criteria.

### 2.2 Specimen Collection and Laboratory Measurements

Fasting peripheral venous blood samples were collected from all patients in the morning and aliquoted into EDTA-anticoagulated tubes, heparin anticoagulant tubes, and plain dried tubes (dry tubes, with one aliquot per tube). EDTA-anticoagulated whole blood was used for complete blood count analysis using an automated hematology analyzer. Blood collected in heparin anticoagulated blood tubes and plain dry tubes was centrifuged at 4,000 rpm for 5 min to separate serum. Serum levels of albumin (ALB), alanine aminotransferase (ALT), aspartate aminotransferase (AST), gamma-glutamyl transferase (GGT), and alkaline phosphatase (ALP) were measured using a Hitachi 7,180 automated biochemical analyzer. Alpha-fetoprotein (AFP) levels were measured using a fully automated immunoassay analyzer. All procedures were performed strictly in accordance with the manufacturers' instructions, and all internal quality controls were within acceptable ranges on the day of testing.

Serum HBV DNA was quantified using a commercially available kit from Sansure Biotech (Changsha, China), namely the Hepatitis B Virus Nucleic Acid Quantitative Detection Kit (PCR-Fluorescence Probing), according to the manufacturer's instructions. The kit is approved by the National Medical Products Administration (NMPA) of China. The lower limit of detection of the kit is 10 IU/mL, and the linear range is 15–2 × 10^9^ IU/mL. This kit has been widely used for HBV DNA quantification in previous clinical studies. In the present study, all enrolled patients had serum HBV DNA below the detection limit of the assay (i.e., <10 IU/mL) and were therefore recorded as HBV DNA-negative.

Serum HBV RNA was quantified using a commercially available kit from Rendu Biotechnology (Shanghai, China), namely the HBV RNA Detection Kit (RNA Capture Probe Method), according to the manufacturer's instructions. Detection limit: The lower limit of detection of this assay was determined to be 100 copies/mL. Positivity threshold: Patients with HBV RNA levels at or above the LLOQ (≥100 copies/mL) were considered as to have detectable HBV RNA, which was defined as the “positive” threshold for our subsequent analyses.

### 2.3 Calculation of PNI and MLR

Based on the five-part differential leukocyte counts measured by the impedance method, the Prognostic Nutritional Index (PNI) and the monocyte-to-lymphocyte ratio (MLR) were calculated as follows:

PNI = 10 × serum albumin (g/dL) + 0.005 × total lymphocyte count ( × 10^9^/L), as originally described by Buzby et al.

MLR was calculated as the ratio of monocyte count to lymphocyte count. MLR = MONO/LYM.

Fibrosis-4 (FIB-4) index was calculated as [age × AST]/[platelet count × √ALT].

APRI was calculated as follows: {[AST (U/L)/ULN of AST] × 100}/platelet count (10^9^/L), where ULN was defined as 40 U/L.

### 2.4 Statistical analysis

Statistical analyses were performed using SPSS version 27.0 (IBM Corp., Armonk, NY, USA), and figures were generated using GraphPad Prism version 9.5 (GraphPad Software, San Diego, CA, USA). Categorical variables were presented as *n* (%) and compared between groups using the chi-square test. Continuous variables with normal distribution were expressed as mean ± standard deviation ($\bar{x}\pm s$) and compared using the independent-samples *t*-test. Continuous variables with non-normal distribution were expressed as median (interquartile range) [M (P_25_₅, P_75_)] and compared using the Mann-Whitney *U*-test. A two-sided *P*-value < 0.05 was considered statistically significant. ROC curves were constructed using GraphPad Prism to evaluate the diagnostic value of PNI and MLR for cirrhosis in patients with HBV DNA-negative/RNA-positive CHB. The AUC, sensitivity, specificity, and Youden index were calculated to determine optimal cut-off values. Pearson correlation analysis was used to examine the relationship between PNI and MLR in the cirrhosis group. A nomogram was constructed using R software version 4.4.0 (The R Foundation) with the rms package based on multivariable logistic regression analysis. Calibration curves were developed to assess the model's goodness of fit and the consistency between predicted and observed probabilities. All statistical tests were two-sided, and *P* < 0.05 was considered statistically significant.

Regarding multivariable logistic regression (detailed methodology):

#### 2.4.1 Variable selection

Candidate variables with a *P*-value < 0.10 in univariable analysis were screened for initial inclusion in the multivariable logistic regression model. To avoid overfitting, we also employed a backward stepwise elimination procedure based on the Akaike Information Criterion (AIC), retaining variables with a *P*-value < 0.05 in the final model to ensure the rationality of variable selection.

#### 2.4.2 Assessment of multicollinearity

To ensure the stability of the regression coefficients, we assessed multicollinearity among all independent variables using the variance inflation factor (VIF). Variables with VIF > 5 were considered to indicate significant collinearity and were excluded or combined. In our final model, all VIF values were less than 2.0, indicating no substantial multicollinearity.

Variables with *P* < 0.05 in multivariate logistic regression were defined as independent predictors. Variables with marginally significant associations (0.05 ≤ *P* < 0.10) and proven clinical relevance were also retained in the nomogram after assessment of multicollinearity.

Model performance and goodness-of-fit: The discriminative ability of the model was evaluated using the area under the receiver operating characteristic curve (AUC). Calibration was evaluated using the Hosmer-Lemeshow goodness-of-fit test and a *P*-value greater than 0.05 was considered indicative of acceptable calibration.

## 3 Results

### 3.1 Baseline characteristics and comparison of PNI and MLR levels between patients with HBV DNA-negative/RNA-positive CHB with and without cirrhosis

Comparison of clinical data between the observation group (with cirrhosis) and the control group (without cirrhosis) revealed no statistically significant differences in age, sex, ALT, ALP, or AFP levels between the two groups (all *P* > 0.05), indicating baseline comparability.

Compared with the control group, the observation group exhibited significantly lower levels of inflammatory markers (white blood cell count, lymphocyte count, and monocyte count) and nutritional markers (TP, ALB, and PA), while AST and GGT levels were significantly higher (all *P* < 0.05). Although these differences reached statistical significance, the mean and median values of these parameters remained within the normal reference ranges in both groups.

Further comparison of PNI and MLR, which were calculated based on serum albumin levels and routine blood count results, revealed that the observation group had significantly lower PNI levels and significantly higher MLR, FIB-4 and APRI levels than the control group (all *P* < 0.001), as shown in Figure 1 and Table 1. In the present cohort, most patients with liver cirrhosis exhibited APRI values below 1.0. The diagnostic performance of APRI was relatively unsatisfactory in this population, which may be attributed to normalized transaminase levels in patients receiving long-term antiviral therapy.

**Figure 1:**
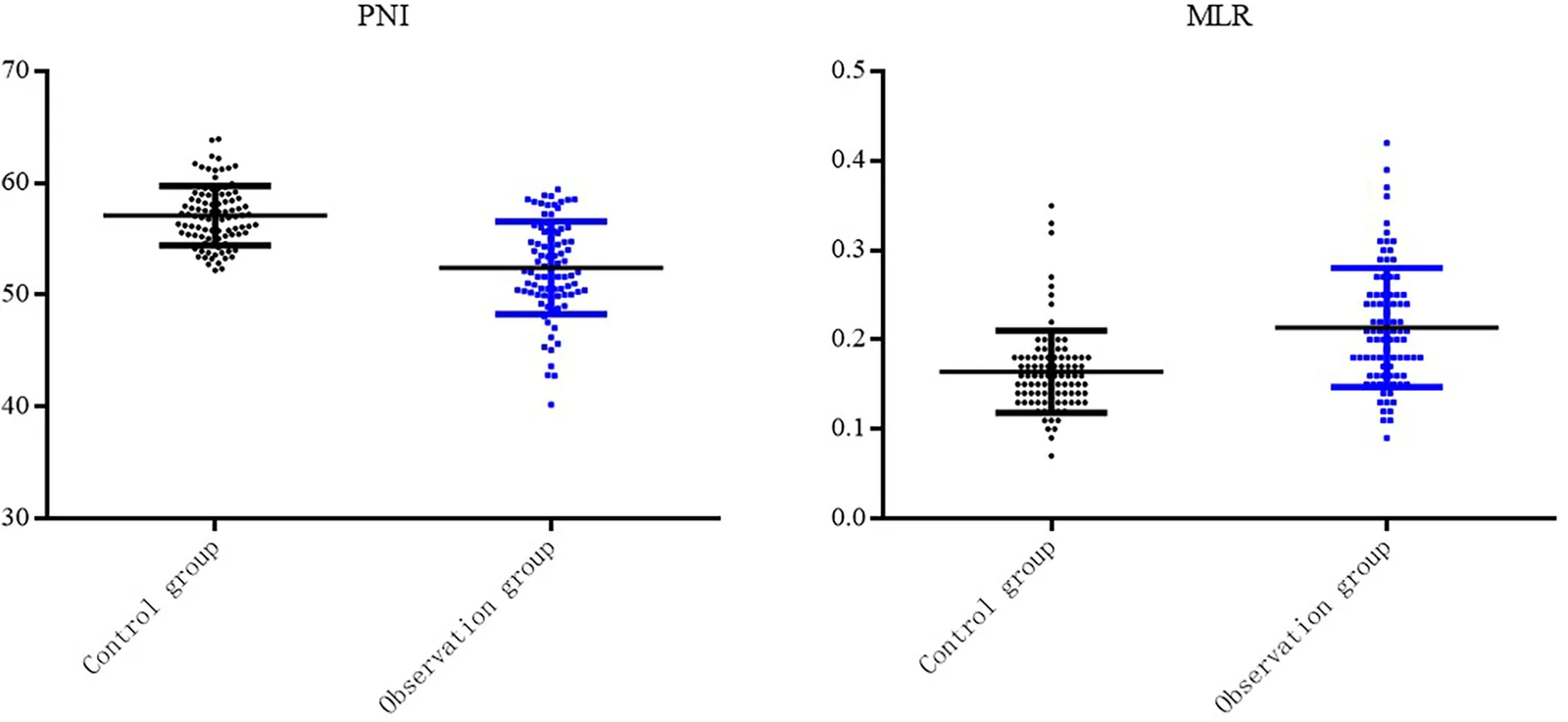
Comparison of PNI and MLR levels between the observation group and the control group.

**Table 1:** Comparison of clinical characteristics and PNI/MLR levels between the observation group and control group.

Index	Control group (*n* = 96)	Observation group (*n* = 87)	*t/χ^2^/z* value	*P* value
Age (years)	52.98 ± 6.99	54.83 ± 9.57	−1.502	0.135
Sex (male/female)	64/32	49/38	2.068	0.150
WBC (×10^9^/L)	5.44 ± 1.26	4.61 ± 1.04	4.825	<0.001
NEU (×10^9^/L)	2.95 ± 0.86	2.78 ± 0.71	1.441	0.151
LYM (×10^9^/L)	2.04 ± 0.49	1.42 ± 0.46	8.859	<0.001
MONO (×10^9^/L)	0.32 ± 0.08	0.29 ± 0.09	2.917	0.004
TP (g/L)	76.48 ± 3.23	74.89 ± 5.07	2.558	0.011
ALB (g/L)	46.88 ± 2.18	45.34 ± 3.31	3.750	<0.001
PA (mg/dL)	23.12 ± 4.55	19.00 ± 5.48	5.550	<0.001
ALT (U/L)	22 (17, 29)	22 (18, 33)	0.442	0.990
AST (U/L)	21 (18, 25)	24 (19, 30)	1.531	0.018
GGT (U/L)	21 (16, 27)	24 (18, 46)	1.395	0.041
ALP (U/L)	76 (64, 90)	80 (67, 100)	0.711	0.693
AFP (ng/mL)	2.49 (1.94, 3.33)	2.25 (1.69, 3.00)	1.085	0.190
HBV RNA (copies/mL)	1,380 (219, 210,000)	724 (269, 5,950)	−1.453	0.146
PNI	57.10 ± 2.65	52.44 ± 4.15	9.142	<0.001
MLR	0.16 (0.14, 0.18)	0.21 (0.16, 0.25)	2.897	<0.001
FIB-4	1.39 (1.01, 1.80)	2.43 (1.31, 3.54)	3.108	<0.001
APRI	0.28 (0.23, 0.38)	0.49 (0.31, 0.85)	2.630	<0.001

TP, total protein; ALB, albumin; PA, prealbumin; ALT, alanine aminotransferase; AST, aspartate aminotransferase; GGT, gamma-glutamyl transferase; ALP, alkaline phosphatase; AFP, alpha-fetoprotein; PNI, Prognostic Nutritional Index; MLR, monocyte-to-lymphocyte ratio. Normally distributed continuous variables are expressed as mean ± standard deviation ($\bar{x}\pm s$ ); non-normally distributed continuous variables are expressed as median (interquartile range) [M (P_25_, P_75_)].

### 3.2 Correlation analysis of PNI, MLR, and other indicators with cirrhosis in patients with HBV DNA negative/RNA positive chronic hepatitis B

Pearson correlation analysis revealed a significant negative correlation between PNI and MLR in patients with HBV DNA-negative/RNA-positive chronic hepatitis B who developed cirrhosis (*r* = −0.484, *P* < 0.001). Although the correlation coefficient indicated a moderate negative correlation, the association was highly statistically significant, as shown in Table 2 and Figure 2.

**Table 2:** Correlation of PNI and MLR with inflammatory-nutritional markers and liver function parameters in the observation group.

Variable	MLR		PNI	
	*r* value	*P* value	*r* value	*P* value
Age (years)	0.144	0.182	−0.197	0.068
WBC (×10^9^/L)	−0.288	0.007	0.510	<0.001
NEU (×10^9^/L)	−0.174	0.107	0.358	0.001
LYM (×10^9^/L)	−0.486	<0.001	0.606	<0.001
MONO (×10^9^/L)	0.424	<0.001	0.144	0.182
TP (g/L)	−0.346	0.001	0.326	0.002
ALB (g/L)	−0.267	0.012	0.831	<0.001
PA (mg/dL)	−0.393	<0.001	0.310	0.004
ALT (U/L)	0.206	0.056	−0.249	0.020
AST (U/L)	0.257	0.016	−0.465	<0.001
GGT (U/L)	0.187	0.083	−0.176	0.103
ALP (U/L)	0.182	0.091	−0.321	0.002
AFP (ng/mL)	−0.243	0.023	0.133	0.220
PNI	−0.484	<0.001	1	–
MLR	1	–	−0.484	<0.001

“–” indicates no data available. TP, total protein; ALB, albumin; PA, prealbumin; ALT, alanine aminotransferase; AST, aspartate aminotransferase; GGT, gamma-glutamyl transferase; ALP, alkaline phosphatase; AFP, alpha-fetoprotein; PNI, prognostic nutritional index; MLR, monocyte-to-lymphocyte ratio.

**Figure 2:**
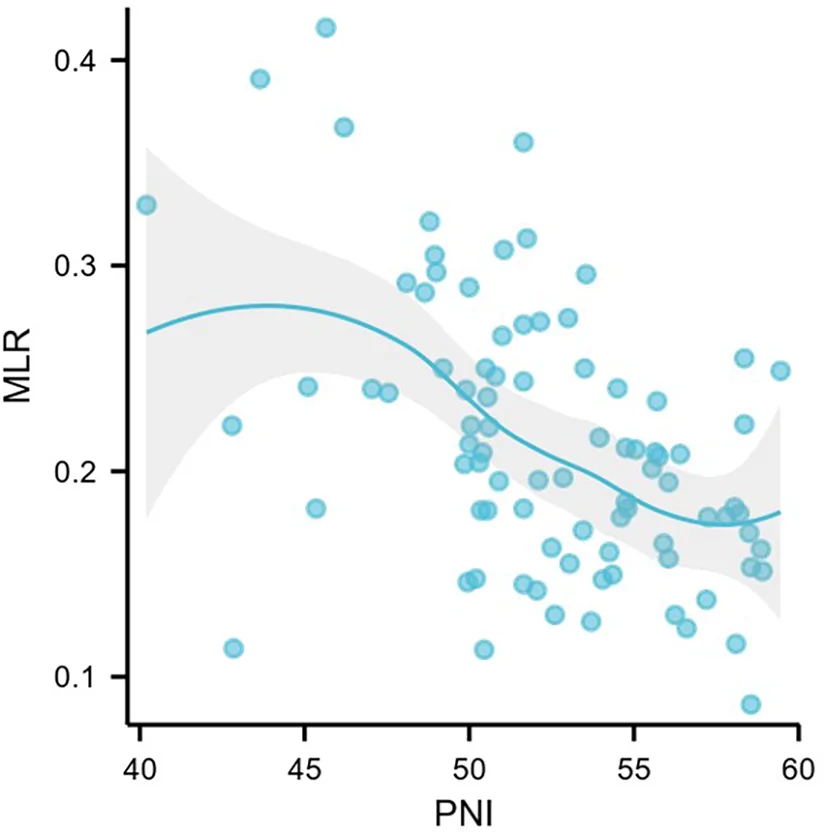
Scatter plots showing the correlation of PNI and MLR with concomitant cirrhosis in patients with HBV DNA-negative/RNA-positive chronic hepatitis B.

### 3.3 Diagnostic performance of PNI and MLR for cirrhosis in patients with HBV DNA-negative/RNA-positive chronic hepatitis B

Using chronic hepatitis B patients without cirrhosis as the control group, we evaluated the diagnostic value of PNI and MLR for concomitant cirrhosisin patients with HBV DNA-negative/RNA-positive chronic hepatitis B. As shown in Table 3 and Figure 3, the areas under the ROC curve (AUC) of PNI and MLR for diagnosing cirrhosis were 0.821 and 0.743, with sensitivities of 95.8% and 85.4%, respectively (both *P* < 0.001). The AUC of FIB-4 and APRI for diagnosing liver cirrhosis was 0.733 and 0.735. These findings indicate that both PNI and MLR demonstrate high sensitivity for diagnosing cirrhosis in patients with HBV DNA-negative/RNA-positive chronic hepatitis B, and may serve as sensitive diagnostic biomarkers to assist clinicians in the early identification of cirrhosis risk.

**Table 3:** Diagnostic performance of PNI and MLR for cirrhosis in patients with HBV DNA-negative/RNA-positive chronic hepatitis B.

Variable	AUC	95%CI	*P* value	Cut-off	Sensitivity	Specificity	Youden index
PNI	0.821	0.760–0.882	<0.001	53.15	0.958	0.563	0.521
MLR	0.743	0.670–0.816	<0.001	0.195	0.854	0.575	0.429
FIB-4	0.733	0.658–0.809	<0.001	2.105	0.885	0.575	0.46
APRI	0.735	0.661–0.809	<0.001	0.415	0.792	0.598	0.39

PNI, Prognostic Nutritional Index; MLR, monocyte-to-lymphocyte ratio; AUC, area under the curve; CI, confidence interval. Youden index = sensitivity + specificity−1. *P* < 0.05 was considered statistically significant.

**Figure 3:**
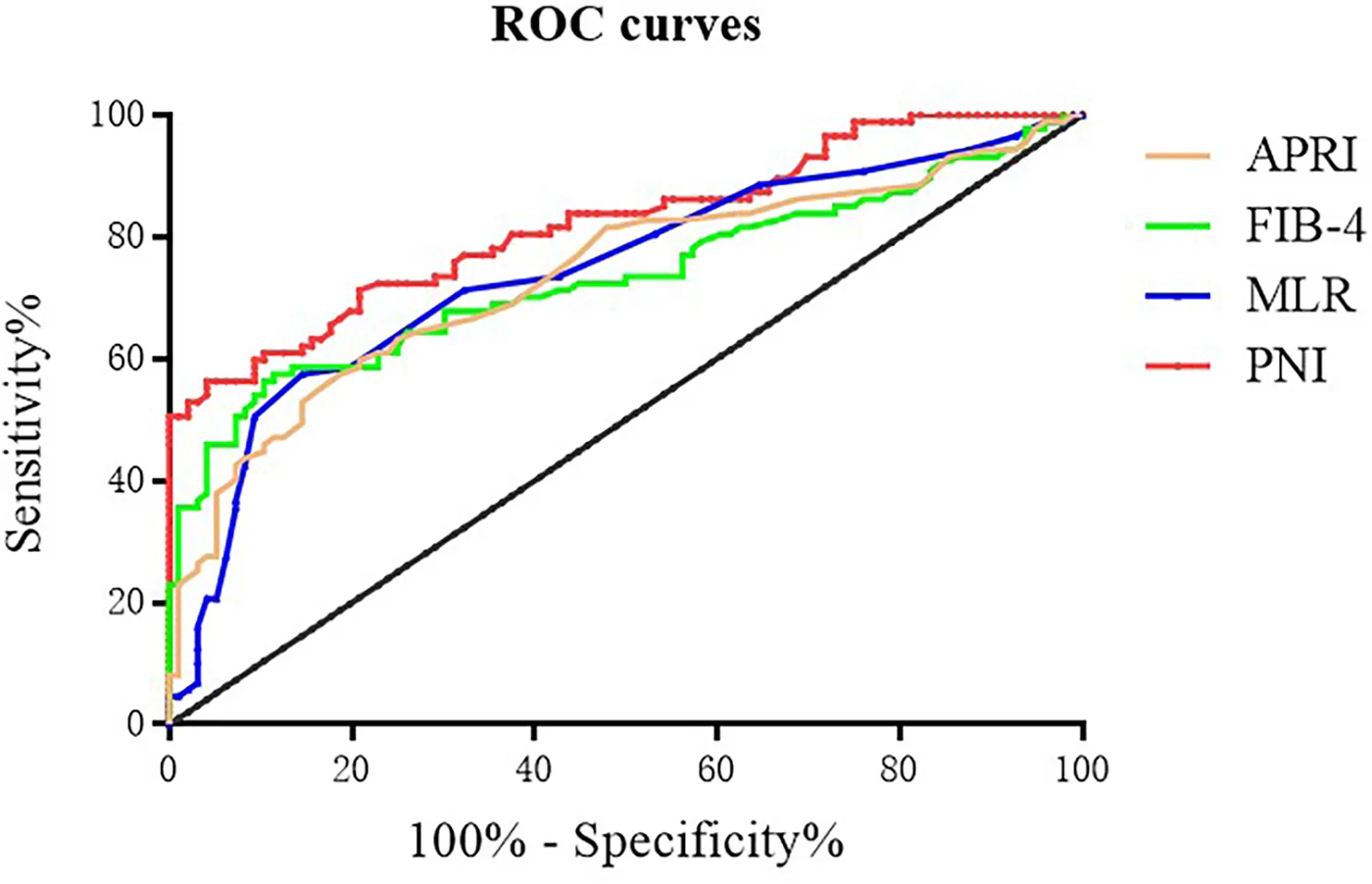
ROC curves of PNI, MLR, FIB-4 and APRI for diagnosing cirrhosis in patients with HBV DNA-negative/RNA-positive chronic hepatitis B.

### 3.4 Univariate and multivariate logistic regression analysis of cirrhosis in patients with HBV DNA-negative/RNA-positive chronic hepatitis B

A multinomial logistic regression model was established to evaluate the relationship between liver function parameters, PNI, MLR, and disease progression in patients with HBV DNA-negative/RNA-positive chronic hepatitis B. The different disease courses (chronic hepatitis B vs. cirrhosis) were set as the dependent variable, with the chronic hepatitis B group serving as the reference. Liver function parameters, PNI, and MLR were included as covariates. The results are summarized in Tables 4,5.

**Table 4:** Univariate logistic regression analysis of liver function parameters, PNI, and MLR for cirrhosis in patients with HBV DNA-negative/RNA-positive chronic hepatitis B.

Index	*β*	SE	Wald	*P* value	OR	95%CI
ALT	0.006	0.010	0.368	0.544	1.006	0.986∼1.027
AST	0.034	0.017	4.183	0.041	1.035	1.001∼1.069
GGT	0.008	0.005	2.819	0.093	1.008	0.999∼1.018
ALP	0.009	0.006	2.234	0.135	1.009	0.997∼1.021
PNI	−0.433	0.068	40.830	<0.001	0.649	0.568∼0.741
MLR	2.069	0.361	32.758	<0.001	7.915	3.897∼16.074

**Table 5:** Multivariate logistic regression analysis of liver function parameters, PNI, and MLR for cirrhosis in patients with HBV DNA-negative/RNA-positive chronic hepatitis B.

Index	β	SE	Wald	*P* value	OR	95%CI
ALT	−0.022	0.021	1.102	0.294	0.979	0.940∼1.019
AST	0.022	0.036	0.367	0.544	1.022	0.952∼1.098
GGT	0.003	0.008	0.112	0.738	1.003	0.988∼1.018
ALP	0.009	0.008	1.174	0.279	1.009	0.993∼1.024
PNI	−0.365	0.073	25. 036	<0.001	0.694	0.601∼0.801
MLR	1.156	0.435	7.064	0.008	3.176	1.354∼7.446

ALT, alanine aminotransferase; AST, aspartate aminotransferase; GGT, gamma-glutamyl transferase; ALP, alkaline phosphatase; PNI, prognostic nutritional index; MLR, monocyte-to-lymphocyte ratio; OR, odds ratio; CI, confidence interval.

MLR showed markedly right-skewed distribution in our cohort. When MLR was included as a continuous variable in univariate logistic regression, quasi-complete separation occurred, resulting in an extremely large odds ratio with an excessively wide 95% confidence interval, which made the estimate unstable. Therefore, ROC curve analysis was performed based on the maximum Youden index to determine the optimal cutoff value, and MLR was converted into high/low groups for univariate screening.

Multivariate multinomial logistic regression analysis revealed that elevated PNI was an independent protective factor against cirrhosis in patients with HBV DNA-negative/RNA-positive chronic hepatitis B (OR < 1, *P* < 0.01), suggesting that higher PNI levels were associated with a reduced risk of cirrhosis. MLR was identified as a risk factor. (OR > 1, *P* < 0.01).

### 3.5 Nomogram Model Incorporating PNI and MLR for Predicting Cirrhosis in Patients with HBV DNA-Negative/RNA-Positive Chronic Hepatitis B

A diagnostic nomogram model was constructed based on the independent factors. Two biomarkers including PNI, MLR, were identified via multivariate logistic regression analysis to construct a predictive model for cirrhosis in patients with HBV DNA-negative/RNA-positive chronic hepatitis B. Internal validation was performed using a calibration curve, as shown in Figures 4,5. Compared with the chronic hepatitis B group, the cirrhosis group exhibited significantly decreased PNI levels and significantly elevated MLR levels, suggesting a close association between these biomarkers and cirrhosisstatus. The nomogram model demonstrated a C-index of 0.829 (95% CI: 0.769–0.889, *P* < 0.05), indicating high accuracy and favorable discriminative ability, with overall statistical significance. The calibration curve showed good agreement between predicted probabilities and actual observed outcomes. These findings suggest that PNI and MLR may serve as promising biomarkers for diagnosing cirrhosis in patients with HBV DNA-negative/RNA-positive chronic hepatitis B, with satisfactory reliability and precision.

**Figure 4:**
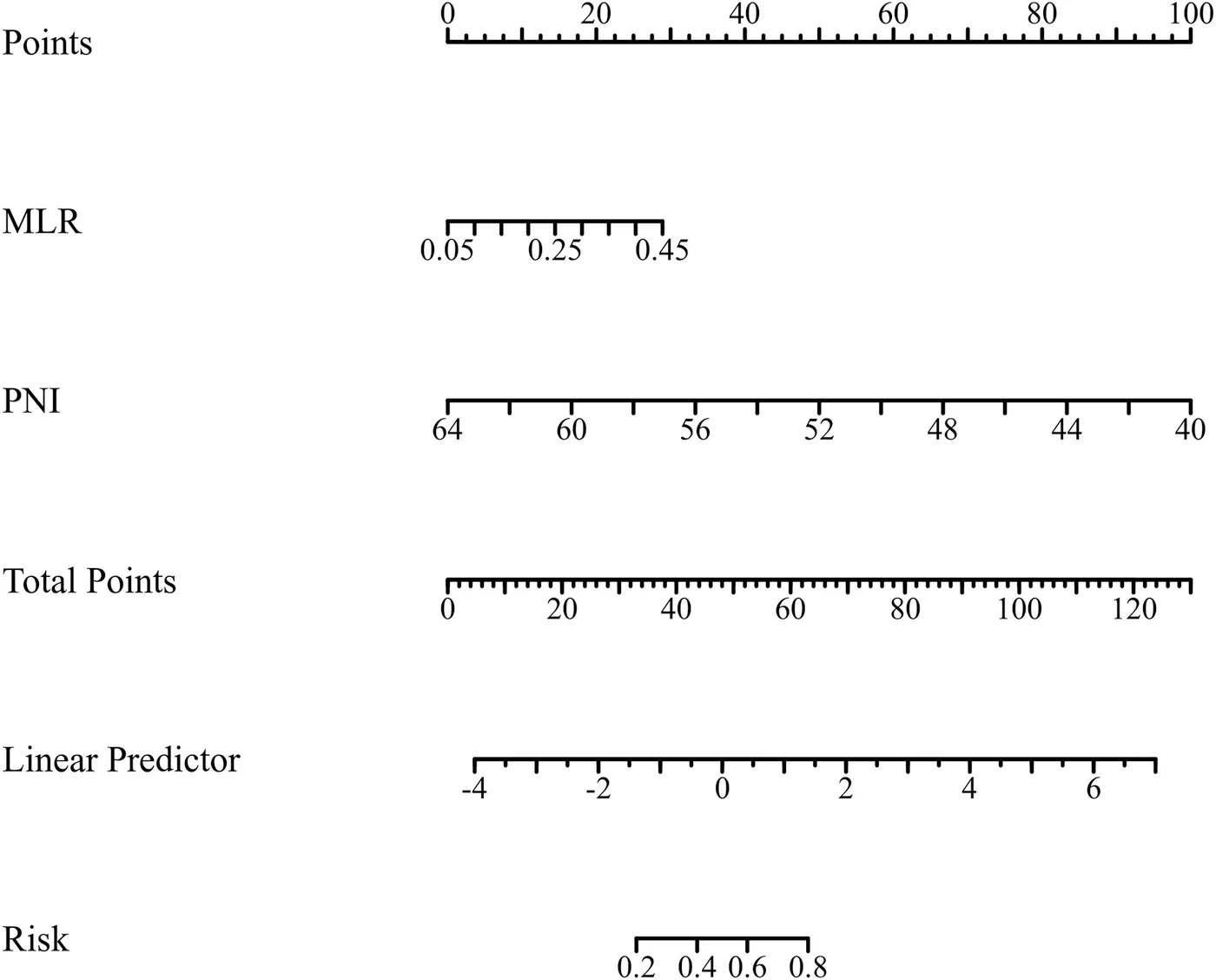
Nomogram model incorporating PNI and MLR for diagnosing cirrhosis in patients with HBV DNA-negative/RNA-positive chronic hepatitis B. PNI, prognostic nutritional index; MLR, monocyte-to-lymphocyte ratio.

**Figure 5:**
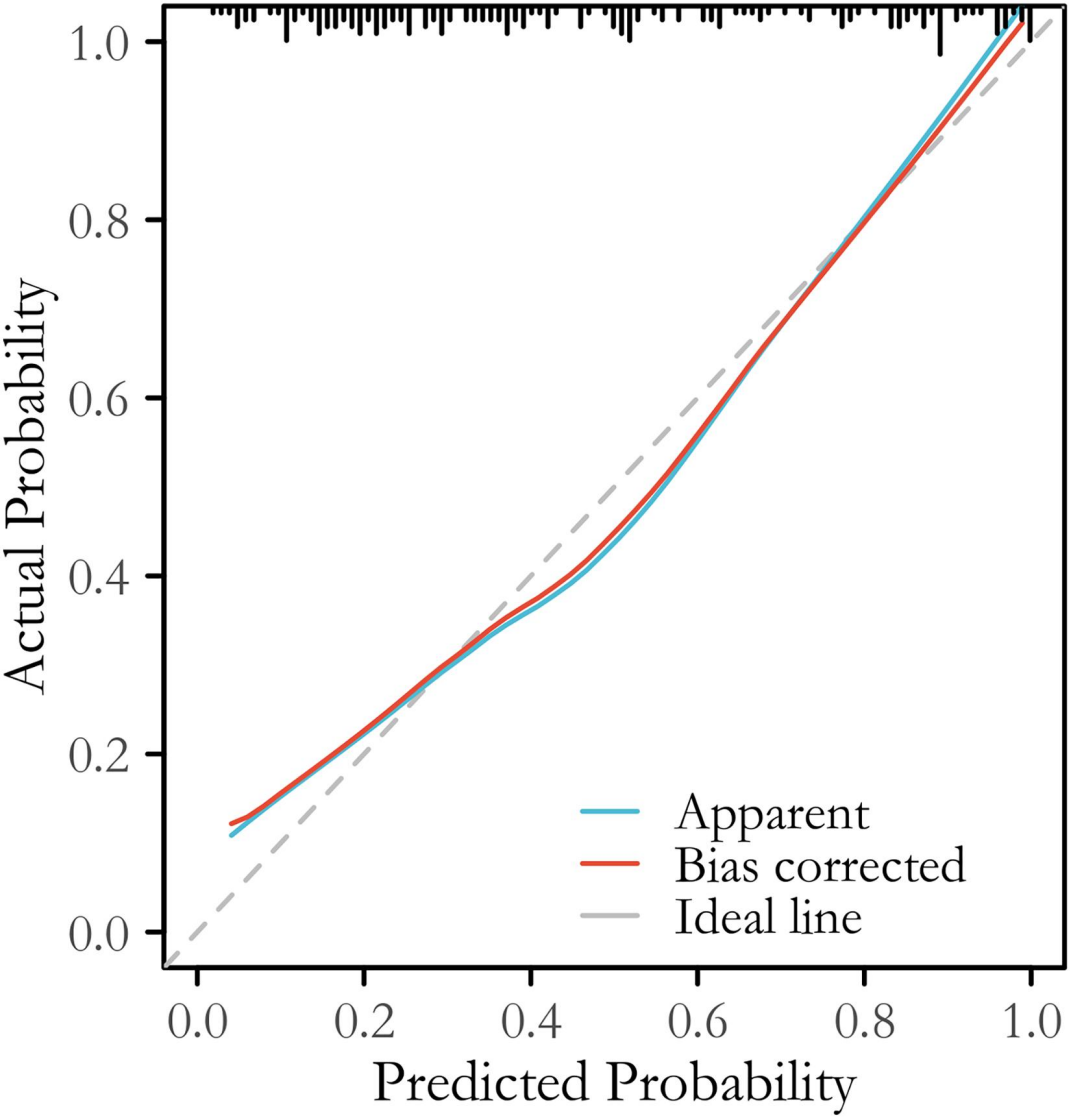
Calibration curve of the nomogram model incorporating PNI and MLR for diagnosing cirrhosis in patients with HBV DNA-negative/RNA-positive chronic hepatitis B.

## 4 Discussion

Hepatitis B virus (HBV) is a hepatotropic DNA virus with a partially double-stranded relaxed circular DNA (rcDNA) genome of approximately 3.2 kb in length (12). HBV remains a major global public health challenge, and its infection-related complications impose an immense disease burden on countless patients worldwide. Consequently, the effective prevention and treatment of HBV have become a top priority in both clinical practice and public health. During the HBV life cycle, following viral entry into hepatocytes, rcDNA is released into the nucleus and converted into covalently closed circular DNA (cccDNA) through the host DNA repair machinery (13). As the transcriptional template for viral replication, cccDNA persists as a stable minichromosome within the nucleus of infected hepatocytes. According to relevant studies, it is the fundamental basis for the difficulty in achieving HBV clearance and the primary cause of virological relapse upon treatment discontinuation (14). HBV RNA, a direct readout of cccDNA transcription, remains detectable even when HBV DNA is undetectable, and thus serves as a reliable biomarker for monitoring viral replication and predicting relapse risk (15). HBV RNA may also be a valuable complementary indicator of cccDNA transcriptional activity in HBV DNA-negative patients (16). The persistent presence of cccDNA within the nucleus of infected hepatocytes constitutes the fundamental reason why HBV infection is difficult to cure and prone to relapse. However, nucleos(t)ide analogues (NAs), which are widely used in clinical practice, target the reverse transcriptase and are incapable of directly eliminating or suppressing the intrahepatic cccDNA pool. This inherent limitation results in virological relapse in a large proportion of patients with chronic hepatitis B or cirrhosis after treatment discontinuation, posing a major challenge in clinical management (17, 18). Currently, conventional biomarkers such as hepatitis B surface antigen (HBsAg) and HBV DNA are routinely used in clinical practice to reflect viral replication status in patients with chronic hepatitis B or cirrhosis. However, when these routine biomarkers fail to provide definitive information, liver biopsy is often recommended to establish a more accurate diagnosis of cirrhosis (19). Nevertheless, as an invasive procedure, liver biopsy carries inherent risks including bleeding and infection, and its availability is constrained by healthcare resources and patient compliance. Consequently, liver biopsy is not universally feasible in all clinical settings. Therefore, there is a pressing clinical need to explore simple, non-invasive, and reliable biomarkers for the diagnosis of cirrhosis.

PNI, proposed by Buzby et al., is a laboratory-based scoring system reflecting immunonutritional status that is calculated from serum albumin concentration and total lymphocyte count. This index is simple to obtain,reproducible and cost-effective (20). PNI has been validated as a valuable prognostic predictor in various diseases, especially in cardiovascular, respiratory, and oncological patients (21). MLR, the monocyte-to-lymphocyte ratio, reflects both innate and adaptive immunity and indicates systemic inflammation (22). PNI and MLR have attracted considerable research interest for assessing of inflammatory and nutritional status, owing to their advantages of simple calculation, easy accessibility and minimal invasiveness. In this study, we demonstrated that the areas under the ROC curve (AUC) of PNI and MLR for diagnosing cirrhosis in patients with HBV DNA-negative/RNA-positive chronic hepatitis B were 0.821 and 0.743, with sensitivities of 95.8% and 85.4%, respectively. These findings indicate that both PNI and MLR possess high sensitivity and may serve as adjunctive inflammatory biomarkers for the clinical diagnosis of cirrhosis in this specific patient population.

In this study, we found that patients with cirrhosis in the HBV DNA-negative/RNA-positive chronic hepatitis B population exhibited significantly lower levels of inflammatory markers (white blood cell count, lymphocyte count, and monocyte count) and nutritional markers (total protein, albumin, and prealbumin), along with significantly higher levels of AST and GGT, compared with those without cirrhosis. These findings suggest that the progression from chronic hepatitis B to cirrhosis in this specific patient population is characterized not only by aggravated hepatocellular injury (as reflected by elevated AST and GGT), but also by activation of systemic inflammatory responses and deterioration of nutritional status (as reflected by decreased inflammatory cells and nutritional markers). These three pathological processes may interact with each other and collectively contribute to disease progression.

The potential interplay among nutritional status, systemic inflammation, and HBV RNA in liver fibrosis progression warrants further elaboration. First, nutritional status, as reflected by serum albumin and prealbumin levels, is not merely a marker of hepatic synthetic function but may actively modulate fibrotic progression. Malnutrition reduces the availability of energy substrates and amino acids essential for hepatocyte regeneration, thereby compromising the liver's capacity to repair injury and rendering it more vulnerable to persistent fibrogenic stimuli. Second, chronic HBV infection is characterized by a sustained low-grade systemic inflammatory state, in which pro-inflammatory cytokines such as tumor necrosis factor-alpha (TNF-α) and interleukin-6 (IL-6) activate hepatic stellate cells and drive extracellular matrix deposition, a key process in fibrosis progression. Third, serum HBV RNA has emerged as a surrogate marker of intrahepatic covalently closed circular DNA (cccDNA) transcriptional activity. Persistent detectability of HBV RNA may reflect active viral transcription, which, in turn, sustains endogenous immune stimulation and may synergize with inflammatory signals to exacerbate fibrogenesis. Importantly, these three factors are likely not independent but rather form a complex, self-reinforcing network: malnutrition exacerbates systemic inflammation, inflammation promotes fibrosis, and progressive fibrosis further impairs liver function, leading to worsening nutritional status. This vicious cycle may accelerate the trajectory of liver disease and underscores the need for integrated therapeutic strategies targeting multiple pathways simultaneously.

In this study, we observed that although the mean/median values of inflammatory markers (white blood cell count, lymphocyte count, and monocyte count) and nutritional markers (TP, ALB, and PA) in cirrhotic patients with HBV DNA-negative/RNA-positive chronic hepatitis B remained within the normal reference ranges, AST and GGT levels were significantly elevated compared with those in non-cirrhotic patients. When these conventional parameters still fall within the “normal” range, the aberrant changes in PNI and MLR become particularly clinically relevant. Compared with non-cirrhotic patients, the cirrhosis group exhibited significantly decreased PNI levels and significantly elevated MLR levels. Furthermore, multivariate logistic regression analysis showed that decreased PNI was associated with an increased risk of cirrhosis in patients with HBV DNA-negative/RNA-positive chronic hepatitis B (OR < 1, *P* < 0.01). MLR was identified as a risk factor (OR > 1, *P* < 0.01). These findings suggest that PNI and MLR may serve as early warning indicators for identifying individuals at high risk of cirrhosis, even when routine laboratory parameters have not yet deviated significantly from the normal range. Low PNI is a well-established predictor of poor prognosis in various diseases, including cardiovascular, oncological, respiratory, and hepatic disorders (23). Therefore, decreased PNI levels in patients with HBV DNA-negative/RNA-positive chronic hepatitis B who develop cirrhosis may indicate severe disease status, ongoing viral replication activity, and a high risk of relapse. Compared with non-cirrhotic patients, those with cirrhosis exhibited lower absolute counts of both monocytes and lymphocytes, with a more pronounced decline in lymphocytes, resulting in an elevated MLR. This indirectly suggests that alterations in adaptive immunity may predominate in the progression to cirrhosis in this specific patient population. The AUCs for PNI, MLR, FIB-4, and APRI were [0.821], [0.743], [0.733], and [0.735], respectively. In patients with HBV DNA-negative/RNA-positive chronic hepatitis B, the diagnostic performance of the prognostic nutritional index (PNI) and monocyte-to-lymphocyte ratio (MLR) for cirrhosis was superior to that of the established non-invasive fibrosis scores, FIB-4 and APRI. We believe these additional analyses convincingly demonstrate that PNI and MLR offer incremental clinical value beyond the widely used FIB-4 and APRI scores. These findings suggest that PNI and MLR may serve as more sensitive and reliable surrogate markers for cirrhosis in this specific patient subgroup, where conventional fibrosis indices may be less informative. We provided mechanistic insights: why these markers may outperform traditional scores in this setting—namely, that FIB-4 and APRI rely heavily on transaminase and platelet dynamics, which are often normalized or minimally altered when HBV DNA is suppressed but HBV RNA persists, whereas PNI and MLR capture nutritional-immune status and systemic inflammation, which are more directly linked to cirrhosis progression. We respectfully believe that this targeted, population-specific validation—addressing a real-world clinical dilemma with an existing, widely available tool—constitutes substantial added value, as it directly informs clinical decision-making in a patient group that lacks evidence-based guidance.

A critical pathophysiological distinction exists between HBV DNA and HBV RNA as virological markers. HBV DNA largely reflects active virion production from intrahepatic covalently closed circular DNA (cccDNA) and correlates closely with the intensity of hepatocellular injury, as evidenced by its association with ALT/AST elevations and platelet dynamics via hypersplenism and bone marrow suppression (24, 25). By contrast, persistent serum HBV RNA—particularly pregenomic RNA—indicates ongoing transcriptional activity from cccDNA or integrated HBV DNA, even when viral replication is suppressed at the DNA level. This distinction has profound clinical implications. In HBV DNA-positive patients, high-level viremia drives robust, overt cytopathic inflammation that directly affects transaminases and platelets—the core components of FIB-4 and APRI. However, in patients who are HBV DNA-negative but HBV RNA-positive (a state frequently encountered in those with incomplete virological suppression or in the “grey zone” of chronic infection), the inflammatory pattern shifts from cytopathic to chronic, smoldering, and immune-mediated. In this state, transaminases often normalize or fluctuate narrowly, substantially reducing the dynamic range and discriminative capacity of FIB-4 and APRI. Platelet counts, moreover, are influenced by multiple extrahepatic determinants beyond portal hypertension, further limiting their specificity. It is precisely this unique inflammatory milieu—rather than overt hepatocellular injury—that preferentially perturbs the immuno-nutritional axis, which is directly captured by PNI and MLR.

We propose three interconnected biological pathways to explain how persistent HBV RNA drives these alterations. First, regarding immune activation and monocyte dysfunction, persistent HBV RNA acts as a pathogen-associated molecular pattern (PAMP) that can be recognized by Toll-like receptors (TLR3, TLR7/8) on monocytes and Kupffer cells, leading to sustained innate immune activation (25, 26). This results in monocytosis, relative lymphopenia, and altered lymphocyte subsets—manifested as an elevated MLR. This pathway is substantially attenuated when HBV RNA is cleared. Second, in terms of nutritional-metabolic derangement, chronic low-grade inflammation driven by persistent viral transcription promotes a systemic catabolic state and suppresses hepatic albumin synthesis. Albumin, a negative acute-phase reactant, is preferentially downregulated under this sustained inflammatory milieu, a process further compounded by HBV RNA-associated endoplasmic reticulum (ER) stress and impaired hepatocyte metabolic function (27). Collectively, these mechanisms contribute to a decline in PNI. Third, and critically, this immuno-nutritional disturbance is qualitatively distinct from the HBV DNA-positive state. In HBV DNA-positive patients, high viremia drives direct cytopathic effects and robust immune-mediated hepatocyte lysis, leading to marked transaminase elevation and thrombocytopenia—alterations that are well-captured by FIB-4 and APRI. However, once DNA is suppressed but RNA persists, the inflammatory pattern transitions to a non-cytopathic, immune-modulatory state. This lower-intensity but more sustained inflammation preferentially depletes nutritional reserves and skews myeloid-lymphoid homeostasis, rendering conventional liver enzyme-based indices less sensitive while making PNI and MLR more informative. This mechanistic distinction provides a plausible explanation for why HBV RNA positivity remained an independent risk factor for cirrhosis even after adjusting for HBV DNA in our multivariate analysis, and why PNI and MLR outperformed FIB-4 and APRI in discriminating cirrhotic from non-cirrhotic patients.

These findings further provide a clear direction for subsequent studies comparing CD4⁺/CD8⁺ T-cell ratios between patients with HBV DNA-negative/RNA-positive chronic hepatitis B and those with cirrhosis. In summary, changes in PNI and MLR levels may serve as potential serum biomarkers for the diagnosis and prognostic assessment of cirrhosis in patients with HBV DNA-negative/RNA-positive chronic hepatitis B, with promising clinical applicability.

Several limitations of this study should be acknowledged. First, owing to the retrospective cross-sectional design, our findings can only reveal statistical associations between biomarker levels and cirrhosis status, rather than establishing causal relationships or temporal precedence. Therefore, all results should be interpreted as correlational findings rather than predictive evidence. Second, the sample size was relatively small and derived from a single center, which may limit the generalizability of our conclusions. Third, the specificity of both biomarkers was relatively modest (56.3% and 57.5%, respectively), which may increase the risk of false-positive results and limit their utility as standalone screening tools in clinical practice. Therefore, these biomarkers should be interpreted with caution and are best used in combination with other clinical parameters or imaging findings. Fourth, the proposed nomogram has not yet been externally validated in an independent cohort, and its generalizability to broader patient populations remains uncertain. Multi-center prospective studies are warranted for external validation. Fifth, we must honestly admit that due to the retrospective nature of this study and the incomplete documentation in the medical records from which data were extracted, we are unable to provide a complete or reliable breakdown of antiviral regimens and treatment durations for all enrolled patients. Several key confounding factors, including antiviral treatment duration, HBeAg status, and HBV RNA levels themselves, may have influenced our findings. The heterogeneity in treatment duration may attenuate the association between HBV RNA and fibrosis, while the inability to perform HBeAg-stratified subgroup analyses due to sample size limitations may have masked important differences. The complex interplay among these factors may introduce residual confounding that cannot be fully addressed by multivariate adjustment alone. Prospective cohort studies with larger sample sizes and better matching strategies are needed to further validate our findings and to explore the potential role of these biomarkers in longitudinal risk stratification. These limitations provide clear directions for future research: larger, multicenter prospective studies are warranted to further validate the diagnostic value of PNI and MLR, and to explore whether nutritional intervention strategies guided by these biomarkers could improve clinical outcomes in this patient population.

Despite the inherent limitations of our study, including the modest specificity of the biomarkers examined, our findings contribute to the growing body of evidence supporting the utility of non-invasive biomarkers in the precision medicine era. Scarlata et al. (28) recently conducted a bicentric retrospective study of 236 cirrhotic patients, evaluating multiple non-invasive biomarkers and clinical scores—including Child–Pugh score, PDW, RPR, and MELD—for predicting cirrhosis-related complications. They reported that the Child–Pugh score achieved the highest overall accuracy (AUC = 0.667) with high specificity (85.42%) but low sensitivity (37.97%), while PDW and RPR showed modest predictive ability for varices (AUC = 0.594), and MELD performed best for hepato-renal syndrome (AUC = 0.758). This supports our conclusion that the biomarkers we evaluated, despite their moderate specificity, may still be clinically useful when interpreted in conjunction with other clinical parameters and imaging findings. Moreover, Li et al. (29) highlighted the potential of cost-effective, accessible biomarkers for early risk stratification, emphasizing that tests with modest performance profiles can nonetheless facilitate timely preventive care when integrated into appropriate clinical pathways. These perspectives reinforce our recommendation that future studies should explore multimodal strategies combining our biomarkers with established clinical scores and emerging novel markers to enhance predictive accuracy and inform personalized clinical decision-making. PNI and MLR are objective, routinely available, and low-cost indicators derived from standard complete blood count and biochemical tests. Unlike HBV RNA and HBsAg, which require specialized molecular assays and may not be universally accessible in resource-limited settings, PNI and MLR can be easily calculated from routine laboratory data. In this study, both PNI and MLR were independently associated with cirrhosis-related disease severity, suggesting that they may serve as practical alternatives or complementary markers when HBV RNA or quantitative HBsAg testing is unavailable. Moreover, as composite indices reflecting systemic nutritional and inflammatory status, PNI and MLR may capture host immune and metabolic alterations that are not fully reflected by viral markers alone. This is particularly relevant in cirrhotic patients with negative HBV DNA, in whom viral replication is suppressed but immune and inflammatory dysregulation may persist (30–32). However, we acknowledge that PNI and MLR are non-specific markers and cannot replace HBV RNA or HBsAg for etiological assessment. Rather, they may serve as accessible adjunctive tools for risk stratification in this population. Future prospective studies are needed to directly compare the predictive performance of PNI, MLR, HBV RNA, and HBsAg, and to validate our findings in larger multicenter cohorts.

## Data Availability

The raw data supporting the conclusions of this article will be made available by the authors, without undue reservation.
